# Characteristics of High-Temperature Proton Exchange Membrane Fuel Cells (HT-PEMFCs) Based on Novel Structures on Electrode Surfaces

**DOI:** 10.3390/ma18061232

**Published:** 2025-03-10

**Authors:** Tongbo Qiang, Weitao Zhang, Qilong Wu, Chaoling Han

**Affiliations:** 1 Luneng New Energy (Group) Co., Ltd., Qinghai Branch, New Hualian International Center, No. 61 Wusi West Road, Xining 810001, China; 202361207171@njtech.edu.cn (T.Q.); 202261207169@njtech.edu.cn (W.Z.); 2 College of Emergency Management, Nanjing Tech University, Nanjing 211816, China; 3 Academy for Advanced Interdisciplinary Studies, Peking University, Beijing 100871, China

**Keywords:** PEMFC, porous electrode, fin design, theoretical model, numerical simulation

## Abstract

The performance of electrodes is the most critical factor determining the output characteristics of high-temperature proton exchange membrane fuel cells (HT-PEMFCs), and the electrode structure directly determines the strength of mass transfer and electrochemical reactions. Therefore, exploring the mechanism of increasing the specific surface area of electrodes is crucial for the design of electrode structures. In this paper, the electrochemical characteristics and mass transport of an HT-PEMFC are investigated based on a three-dimensional single-channel model, and a mathematical model of the fin structure on the electrode surface is established to make comparisons with calculations. The results indicate that the oxygen mole concentration decreases with an increase in fin density. Meanwhile, the fuel cell reaches optimal performance at a low operating voltage and in high fin density conditions. In addition, the output performance of the PEMFC increases with the aspect ratio. Finally, the potential distribution of the simulation results coincides with the theoretical model, and the mechanism of electrode polarization on the performance of fin geometry can significantly support the interpretation of kinetic characteristics obtained from simulations. The research result contributes to the efficient design and preparation of future electrode structures of HT-PEMFCs.

## 1. Introduction

High-temperature proton exchange membrane fuel cells (HT-PEMFCs) are widely used because of their highly dynamic characteristics and favorable water management. Fuel cell performance and defects depend on various aspects such as the geometric structure of the gas channel, transport properties of reactants inside the gas diffusion layer, electrochemical reactions on the surface of porous electrodes, and the selective permeability of the PEM [1,2,3,4,5,6]. Among them, the structure of the electrode surface has the greatest impact on the electrochemical reaction intensity and PEMFC output performance.

The active area of the porous catalytic electrode in terms of geometry structure plays an important role in the oxidation and reduction reaction process [7,8,9]. An analysis of electrode geometry with different shapes and sizes showed a significant enhancement in dye-sensitized solar cells (DSSCs) by optimizing electrode length [10]. Meanwhile, a hollow carbon electrode structure increases the specific surface area remarkably and shortens the diffusion paths of electron transfer [11]. The value of the thickness of the reaction layer is dependent on the surface structure of the diffusion area on the electrode [12]. Similarly, a thin film electrode composed of Pt can enhance the electro-activity of the anode through an optimized fin-like structure [13]. Moreover, the fine surface structure of the electrode can significantly improve the efficiency of unassisted water splitting and remove the limitation of ion transport in supercapacitors by saving the ionic path [14,15,16].

Recent studies have shown that increasing the electrode surface area is one of the effective methods to improve the performance of PEMFCs. A novel architecture with a square chordal catalyst was designed by Ahmar et al. [17], where the chemical reaction taking place on the surface was more efficient. Wu et al. [18,19,20] found that a catalyst layer surface fabricated by a prominence-like structure, combined with an inclined gas channel, can massively improve the cell performance of a PEMFC. Thus, an electrochemical reaction model of the electrode surface was established by experiments or simulations focusing on different shapes of particle geometry to explicate how the structure of the catalyst layer affects cell performance [21,22].

Therefore, electrode structures with high specific surface areas have been intensively studied as a means to increase the density of electrons or ion transfer. The nanostructure electrode [23,24] fabrication process with a 3D hierarchical geometry has a greater effective surface area; thus, it can effectively enhance the current density of the cell. The surface projection structure is similar to that of a fin in the heat transfer field, which, similarly, has a significant effect on increasing the heat transfer area and ameliorating heat flux distribution [25,26]. It is reasonable to investigate the electric field theory of the electrode fin surface by developing a model of the fin in the field of heat transfer.

In this article, a three-dimensional electrode model is established by analyzing the commonly used fin-shaped electrode structures in experiments. By studying parameters such as different fin densities and aspect ratios, the optimal structure can be obtained. This provides a theoretical basis for future experimental electrode preparation.

## 2. Model Definition

**Figure 1:**
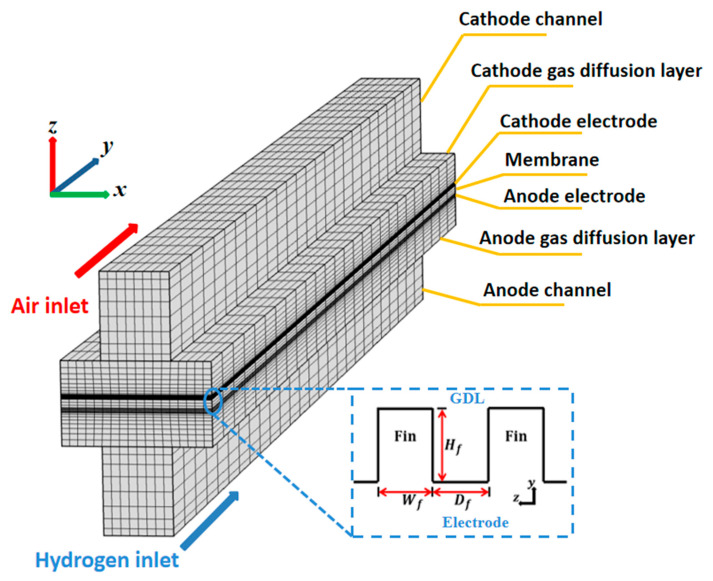
Geometry of PEMFC with a fin-like structure.

In our work, a single-channel model for an HT-PEMFC was selected as the research object. As shown in Figure 1, the gas diffusion layer (GDL) and the porous electrode, also called the catalyst layer (CL), can be regarded as the porous media, and the reactants reach the surface of the electrode through penetration and diffusion inside the porous media. Hydrogen is used as fuel, while air is the oxidizer in the fuel cell system; both of them enter the flow channel from an identical direction. However, in the model optimization of the electrode surface, the CL is designed as a regular fin-like structure, which can be used to investigate the performance of different electrode structures. The fin structure has the same porosity and physical properties as the electrode, and the geometry dimensions of the PEMFC are shown in Table 1 [27,28].

### 2.1. Governing Equations

In order to simplify the simulation process, the mathematical model was simplified based on the guarantee of physical integrity, and the following assumptions were made:

(1) The PEMFC simulation process is in a steady state, with electrical insulation of the shell.
(2) The gaseous components in the simulation are regarded as ideal gases.
(3) Liquid water can be neglected in the HT-PEMFC system.
(4) The flow model inside the gas channel is regarded as laminar.
(5) The porous properties of the GDL and CL are isotropic.
(6) There is a sufficient supply of reactants near the electrode surface.

#### 2.1.1. Continuity Equation

(1) $$\nabla \cdot \left(\rho u\right)=S_{m}$$

where $S_{m}$ represents the mass source terms and ignores the phase change of liquid water. The source term can be simplified to account for the generation or consumption in the redox reaction.

#### 2.1.2. Momentum Equation

The gas flow in the porous media inside the fuel cell is described by the Brinkman equation [29], which is combined with the momentum and mass conservation equations:

(2) $$\left(u\cdot \nabla \right)\frac{u\cdot \rho }{\varepsilon^{2}}=-\nabla P+\nabla \cdot \left[\frac{1}{\varepsilon }\left\{\mu \left(\nabla u+{\left(\nabla u\right)}^{T}\right)\right\}-\frac{2}{3}\mu \left(\nabla u\right)\cdot I\right]+S_{u}$$

where $S_{u}$ is the momentum source term.

#### 2.1.3. Species Transport Equation

The function of the transport of different species in the porous media is based on the Maxwell–Stefan diffusion model [29], as shown in Equations (8) and (9):

(3) $$\rho \nabla \cdot \left(w_{i}u\right)=-\nabla \cdot Q_{i}+R_{i}$$

(4) $$\left\{\begin{array}{l} \sum_{i=1}^{n}w_{i}=1\;\;\;\;\;\;\;\;\;\;\;\;\;\;\;\;\;\;\;\;\;\;\;\;\;\;\;\;\;\;\;\;\;\;\;\;\;\;\;\;\;\;\;\;\;\;\;\;\;\;\;\;\;\;\;\;\;\;\;\;\;\;\;\;\;\;\;\;\;\;\;\;\;\;\;\\ Q_{i}=-\rho w_{i}\sum_{k=1}^{n}{\tilde{D}}_{{ik}_{eff}}d_{k}-\frac{D_{i}^{T}}{T}\nabla T\;\;\;\;\;\;\;\;\;\;\;\;\;\;\;\;\;\;\;\;\;\;\;\;\;\;\;\;\;\;\;\;\;\;\;\\ d_{k}=\nabla x_{k}+\frac{1}{P}\left[\left(x_{k}-w_{k}\right)\nabla P-\rho w_{k}g_{k}+\sum_{i=1}^{n}\rho w_{l}g_{l}\right] \end{array}\right.$$

(5) $${\tilde{D}}_{{ik}_{eff}}=D_{iko}{\left(\frac{T}{T_{0}}\right)}^{1.5}\cdot \varepsilon^{1.5}$$

where $w_{i}$ represents the mass fraction of different components, $Q_{i}$ is the mass flow ratio at the mean velocity for component i, $R_{i}$ denotes the mass rate of consumption for component i, ${\tilde{D}}_{{ik}_{eff}}$ is the effective diffusion coefficient calculated by the Bruggman correlation [30,31], and$\;d_{k}$ stands for the diffusion driving force coefficient for independent components k. The bulk force resulting from the migration of the electric field can be neglected; thus, $g_{k}=0$.

#### 2.1.4. Electrochemical Equation

The electronic and ion transport processes in the electrode surface and electrolyte layers can be expressed by Ohm’s law:

(6) $$-\nabla \cdot \left(\sigma_{eff}^{s}\cdot \nabla \phi_{s}\right)=S_{s}=j_{s}$$

(7) $$-\nabla \cdot \left(\sigma_{eff}^{l}\cdot \nabla \phi_{l}\right)=S_{l}=j_{l}$$

The current density of the electrolyte $j_{l}$ is the same as the current value on the electrode surface $j_{s}$, but in the opposite direction. The electrode reaction kinetics for both the anode and cathode follow the Butler–Volmer equation:

(8) $$j_{loc}^{a}=j_{ref}^{a}{\left(\frac{C_{H_{2}}}{C_{H_{2}}^{ref}}\right)}^{0.5}\left[esp\left(\frac{\alpha_{a}F\left(\phi_{s}-\phi_{l}\right)}{RT}\right)-esp\left(\frac{-\alpha_{c}F\left(\phi_{s}-\phi_{l}\right)}{RT}\right)\right]$$

(9) $$j_{loc}^{c}=j_{ref}^{c}\left(\frac{C_{O_{2}}}{C_{O_{2}}^{ref}}\right)\left[-esp\left(\frac{\alpha_{a}F\left(\phi_{s}-\phi_{l}-E_{eq}\right)}{RT}\right)+esp\left(\frac{-\alpha_{c}F\left(\phi_{s}-\phi_{l}-E_{eq}\right)}{RT}\right)\right]$$

where $C_{H_{2}}^{ref}$ is the reference bulk concentration of $H_{2}$ and $C_{O_{2}}^{ref}$ is the reference concentration of $O_{2}$ correspondingly. The sum of the cathode and anode transfer coefficients $\alpha_{a}$ and $\alpha_{c}$, which represents the reaction strength, is equal to 1.

#### 2.1.5. Electric Conduction Model of Fin-like Structure

Adapting the heat conduction differential equation of a fin in the heat transfer field, the Laplace equation of the electric conduction process [27] on the electrode fin is shown in Equation (10):

(10) $$\frac{\partial^{2}\phi }{\partial x^{2}}+\frac{{\dot{J}}_{s}}{\sigma_{cl}}=0$$

**Figure 2:**
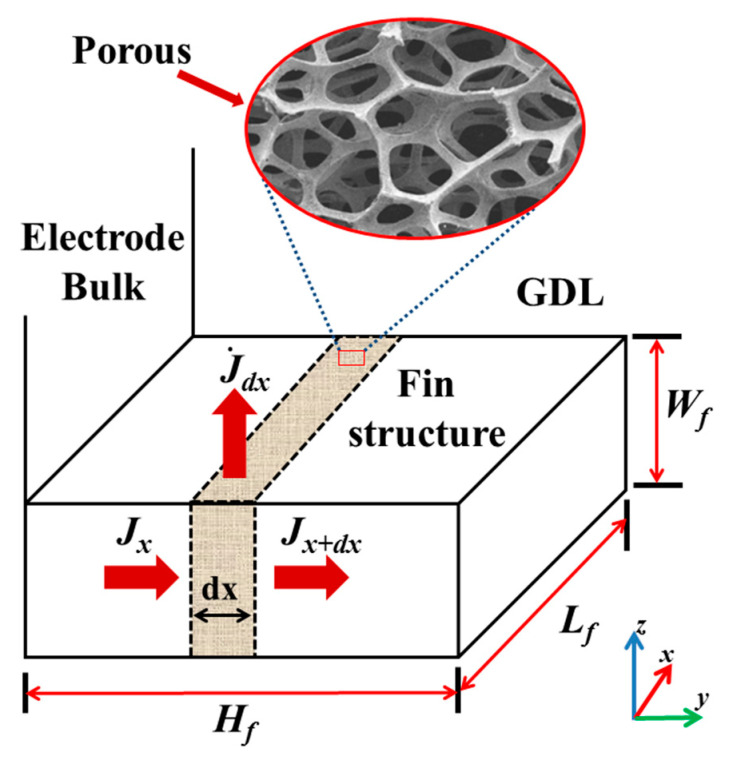
Mechanism model of fin conduction [27].

The voltage changes of the fin can be converted into the source term of the unit volume. As shown in Figure 2, the unit with length dx serves as the calculating microelement, where the specific surface area $\sigma_{A}$ [32,33] of the porous structure is $6\times 10^{6}$ m^−1^ [34,35] in this study. Thus, the surface current of the microelement is as follows:

(11) $$J_{s}=\left(\phi -\phi_{\infty }\right)\sigma_{A}\left(A_{c}dx\right)/R_{s}$$

The effective volume of the porous structure can be expressed in terms of the porosity $\varepsilon_{cl}$. The volume of the microelement is $A_{c}\left(1-\varepsilon \right)dx$, where the source term ${\dot{J}}_{s}$ is deduced as follows:

(12) $${\dot{J}}_{s}=\frac{J_{s}}{A_{c}\left(1-\varepsilon_{cl}\right)dx}$$

(13) $$\frac{{\dot{J}}_{s}}{\sigma_{cl}}=-\frac{\left(\phi -\phi_{\infty }\right)\sigma_{A}}{\left(1-\varepsilon_{cl}\right)\sigma_{cl}R_{s}}$$

Plug Equation (13) into Equation (10) and assume the current is negative where transmitted from the fin to the outside:

(14) $$\frac{d^{2}\phi }{dx^{2}}=\frac{\left(\phi -\phi_{\infty }\right)\sigma_{A}}{\left(1-\varepsilon_{cl}\right)\sigma_{cl}R_{s}}$$

The corresponding boundary conditions can be described as follows:

(15) $$\left\{\begin{array}{c} x=0;\;\phi =\phi_{0}\\ x=H;\;\frac{d\phi }{dx}=0 \end{array}\right.$$

A mathematical model with a second-order non-homogeneous differential equation based on the potential distribution for the electrode fin was established by solving Equations (15) and (16) simultaneous. By introducing the overpotential $\eta_{c}=\phi -\phi_{\infty }-E_{eq}$ for the cathode side as the example, the equation becomes

(16) $$\frac{d^{2}\phi }{dx^{2}}=m^{2}\eta_{c}$$

The boundary conditions in Equation (14) are converted to the following:

(17) $$\left\{\begin{array}{l} x=0;\;\eta_{c0}=\phi_{0}-\phi_{\infty }\\ x=H;\;\;\;\;\;\;\;\;\;\;\;\;\;\frac{d\eta_{c}}{dx}=0 \end{array}\right.$$

where $m=\sqrt{\sigma_{A}/\left[\left(1-\varepsilon_{cl}\right)\sigma_{cl}R_{s}\right]}$ is constant, and the source terms are shown in Table 2. The overpotential distribution along the fin surface in the *y*-direction is obtained by solving Equations (16) and (17):

(18) $$\eta_{c}=\eta_{c0}\frac{cosh\left[m\left(x-H\right)\right]}{cosh\left(mH\right)}$$

### 2.2. Boundary Conditions

The boundary conditions for the calculation are listed in Table 3. In the present study, a non-slip condition is applied to the velocity boundaries, which remain constant at the inlet position of the gas channel (GC). Meanwhile, as shown in Figure 2, all components of the PEMFC, except the GC, have symmetry boundaries along the *z-y* cross-section. The shell boundary of the GC is set as an insulation boundary, and the operating voltages are scanned from 0.9 to 0.1 V in increments of 0.05 V the during calculation testing. The physical parameters used [28,36,37,38] in the simulation are presented in Table 4.

## 3. Results and Discussion

### 3.1. Validation of Simulation Model

**Figure 3:**
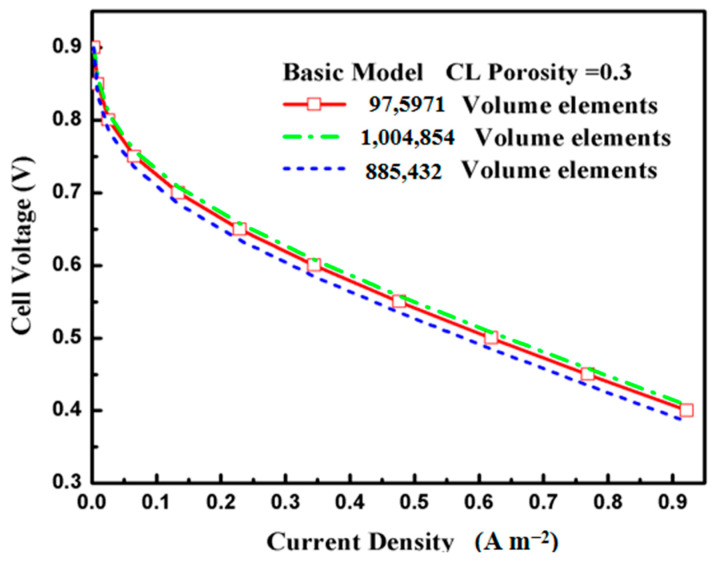
Grid independence test.

In our study, we used a fin-like surface electrode structure, where the distance between two adjacent fins was varied according to four different fin density ratios, with three various aspect ratio structures to investigate the effects of the novel electrode surface structure. Table 5 lists the sizes of the different types of fin cases used in the present study. Based on the original model, for example, three different grids resolutions of 885,432 elements, 1,004,854 elements, and 975,971 elements were created under the same conditions to verify the independence of the mesh. The model polarization curves for different mesh numbers are shown in Figure 3. A grid number of 975,971 was chosen in this simulation, and the model polarization curves for the different mesh numbers shown in the graph are less than 0.06%. Moreover, the grid numbers of the different cases and fin densities chosen in this simulation are shown in Table 6. In order to verify whether the model can predict the PEMFC performance correctly, the experimental values under the same geometric and boundary conditions were compared with the simulation.

**Figure 4:**
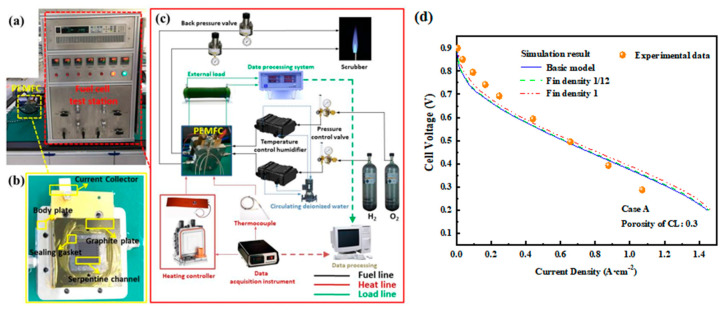
(**a**) PEMFC testing platform; (**b**) assembly process flowchart of PEMFC; (**c**) test system flowchart; (**d**) polarization curves compared with the experimental values.

The polarization curve of a single cell based on the same operating conditions as the simulation experiment was obtained using the fuel cell testing platform at the Academy for Advanced Interdisciplinary Studies, Peking University. A typical PEMFC single-cell test system is shown in Figure 4a. A commercial perfluorosulfonic acid proton exchange membrane, Nafion 212, was used as the electrolyte membrane. The main steps for single-cell preparation are as follows [29,30,31,32]:

(1) Pre-treatment of the Proton Exchange Membrane

First, the Nafion 212 membrane is treated in a 5% hydrogen peroxide solution at 80 °C for 1 h to remove impurities. It is then washed with deionized water and immersed in a 5% H_2_SO_4_ solution for 1 h to ensure full protonation. Afterward, the membrane is washed with deionized water and dried completely in a vacuum drying oven at 40 °C for later use.

(2) Preparation of Catalyst Ink

Catalyst ink with 10% Pt/C is then mixed with a 5% Nafion solution at a 3:1 ratio in isopropanol and ultrasonically dispersed for 1 h using an ultrasonic homogenizer to form a homogeneous catalyst slurry.

(3) Fabrication of Membrane Electrode Assembly (MEA)

A gas diffusion electrode (GDE) is prepared by uniformly coating a hydrophobic carbon paper microporous layer (0.02 m × 0.02 m) with a mixture of Pt/C (loading: 0.5 mg/cm^2^). The membrane electrode assembly (MEA) is then formed by hot pressing two GDEs onto a pretreated proton exchange membrane using a hot press machine at 150 °C, 10 MPa, for 15 min.

(4) Single-Cell Assembly

In the PEMFC, the end plates and bipolar plates serve to support the internal structure and conduct current. The graphite flow field plate functions as a gas channel, with a serpentine flow field design on the inner side. Sealing rings around the cell prevent leakage of reactant gases. The fuel cell is sealed with plastic gaskets and assembled using screws. Before operation, gas is introduced to check the sealing integrity of the system.

(5) Activation of the MEA Before Operation

Before the PEMFC power output test begins, the MEA must be fully activated to ensure that the polymer structure inside the newly prepared proton exchange membrane is sufficiently hydrated and has high ionic conductivity. In this experiment, a constant current and constant voltage alternation method is used. First, the MEA is operated at a constant voltage of 0.3 V for 1 h. Then, a constant current of 1 A is applied, increasing by 0.5 A every 20 min, and maintained for 1 h. These two operating modes are repeated until the voltage reaches or falls below 0.3 V. The hydration process of the proton exchange membrane is facilitated by water generated from the cathodic electrochemical reaction and the humidified reactant gases at the anode and cathode inlets. Additionally, during transitions between constant current and constant voltage operation, nitrogen purging for 2 min is performed to remove excess liquid water from the flow channels, preventing electrode surface flooding due to excessive electrochemical reactions. The activation process is considered complete when the operating current of the test cell remains stable with less than 10% fluctuation over 1 h.

Figure 4b,c show schematic diagrams of the PEMFC single-cell test system. During the experiment, hydrogen at the anode and oxygen at the cathode were released from gas storage tanks, with the pressure regulated by valves and the gas flow controlled by mass flow meters to meet experimental requirements. The reactant gases then entered a temperature-controlled humidifier for humidification. After being heated to the preset temperature by a temperature controller, the gases entered the PEMFC, where oxidation occurred at the anode and reduction occurred at the cathode. The fuel cell body was temperature-controlled by an external heating belt connected to a heating system, combined with a thermocouple temperature measurement system. The generated current was regulated by an external circuit load, which recorded the varying current and voltage data. Finally, the exhaust gases were discharged through an outlet pipeline for combustion and exhaust gas treatment.

As illustrated in Figure 4**,** the polarization curves at different fin densities are all in good agreement with the theoretical values. The difference due to the pore size distribution and porosity of the porous electrodes significantly affects the transfer of gases and ions, and these details may not be accurately captured, leading to concentration polarization errors related to mass transfer.

### 3.2. Oxygen Concentration Distribution

**Figure 5:**
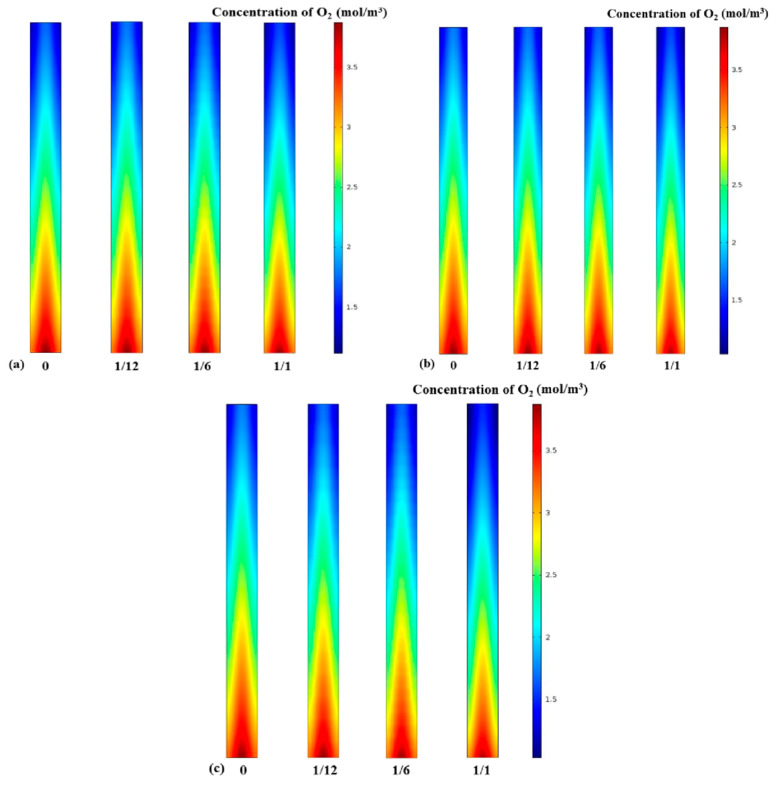
Molar concentration of oxygen: (**a**) Case A with different fin densities; (**b**) Case B with different fin densities; (**c**) Case C with different fin densities.

The performance of a fuel cell is determined by the reactant species participating in the reaction; therefore, the variation in the concentration of the reactants is considered an important part of this study [6]. The water that exists in the system can be regarded as gaseous under the condition of a high operating temperature; thus, the obstacle effect of liquid water in the gas diffusion layer can be neglected [27]. Figure 5 shows the influence of different fin densities on the molar concentration of oxygen at the interface between the PEM and the cathode GDL. At an operating cell voltage of 0.4 V, it is found from Figure 5 that the distribution of oxygen molar concentration depends on the electrode fin density, position, and the aspect ratio of the fins. Clearly, the oxygen concentration at the inlet side of the air is higher than downstream because oxygen continues to be consumed by reduction reactions. Also, it can be observed in all three cases that along the length of the channel, the saturation of oxygen at the median position is higher than at the bilateral positions, as the diffusion effect at the underside of the GC is better than at the two bilateral positions [36]. Figure 5a,c show that under the same fin density, more oxygen was consumed in Case C than in other cases. Furthermore, these contours disclose that the oxygen mole concentration decreased as the number of electrode fins increased, which is mainly due to the fin-made electrode having a larger reaction surface area.

### 3.3. Kinetic Characteristics of Electrochemical Reaction

**Figure 6:**
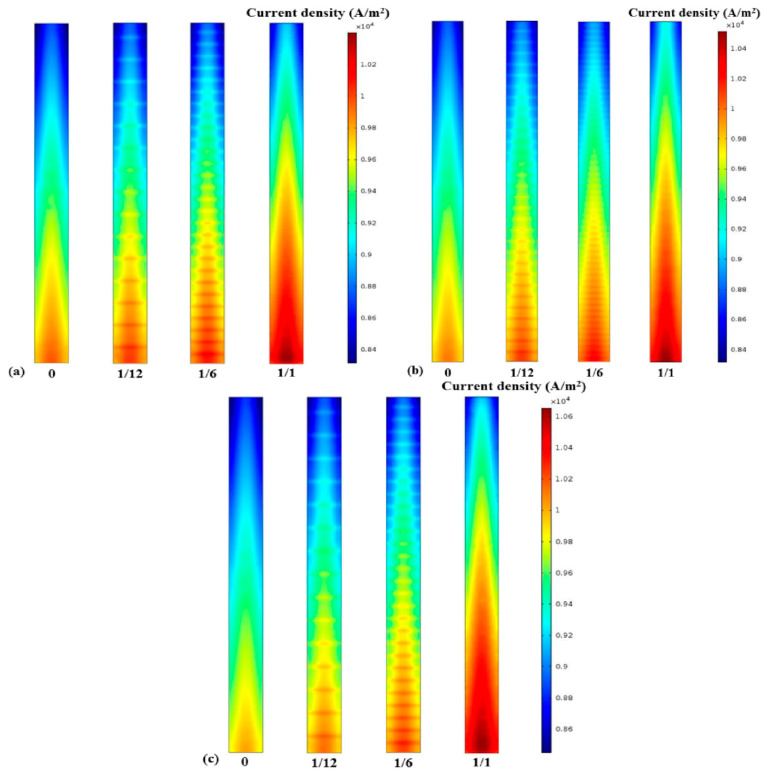
Current density of (**a**) Case A with different fin densities; (**b**) Case B with different fin densities; (**c**) Case C with different fin densities.

**Figure 7:**
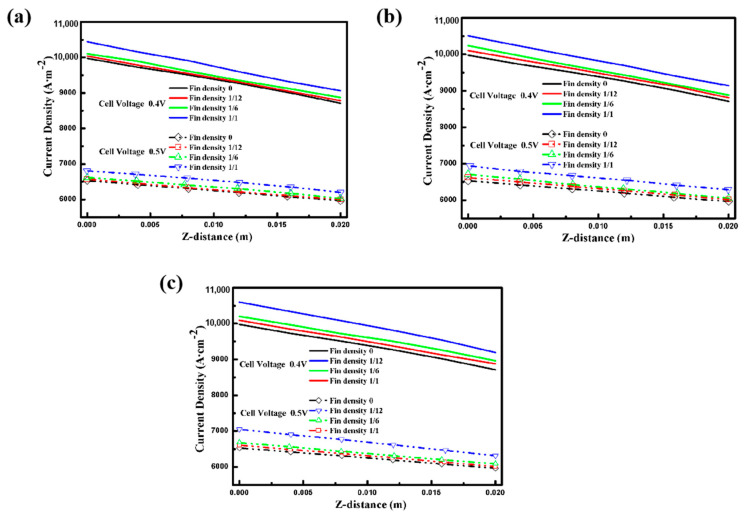
Current density at the center of the PEM with different cell voltages: (**a**) Case A with different fin densities; (**b**) Case B with different fin densities; (**c**) Case C with different fin densities.

Current density and cell potential, as well as reactant concentration, are the key factors that affect the performance of a fuel cell. Figure 6 shows the current density contours for the different aspect ratios and fin densities of the electrode fins. A similar trend for current density in the intermediate layer of the PEM is clearly seen. The current density of the upstream reactant is higher than that of the downstream one, no matter what the electrode structure is. The current density reaches the maximum at the mid-position of the proton exchange membrane along the axis of the length direction, which is similar to the concentration distribution of oxygen. It is evident from Figure 6a,c that certain electrode geometries, especially those with a larger specific surface area and more intensive fin density, can obtain a higher current density. Therefore, to elucidate the influence of electrode structure on current density under different cell voltage conditions [10], Figure 7 depicts the current density at the center line of the PEM interlayer under different conditions. In accordance with previous discussions, the electrochemical reaction intensity is limited gradually along the inlet direction of the reactant due to the constant consumption of oxygen, so the current density decreases, following the same trend as the reactant concentration [12]. Moreover, the results indicate that the performance of the cell increases with the larger contact area between the electrode and the GDL. Thus, the cell performance with an electrode fin density of up to 1 was the best, as shown in Figure 7c. Furthermore, although the current density distribution trend is the same at cell voltages of 0.4 V and 0.5 V, the overall performance is lower at 0.5 V due to the weaker electrochemical reaction rate.

**Figure 8:**
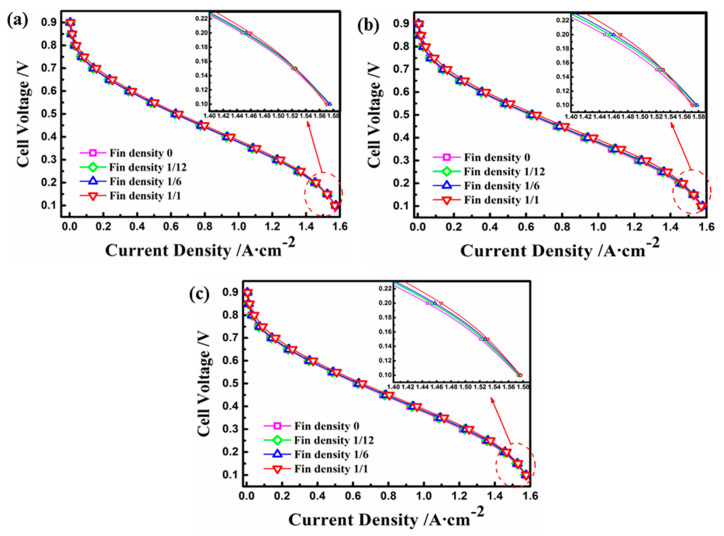
Polarization curves of different electrode fins: (**a**) Case A with different fin densities; (**b**) Case B with different fin densities; (**c**) Case C with different fin densities.

**Figure 9:**
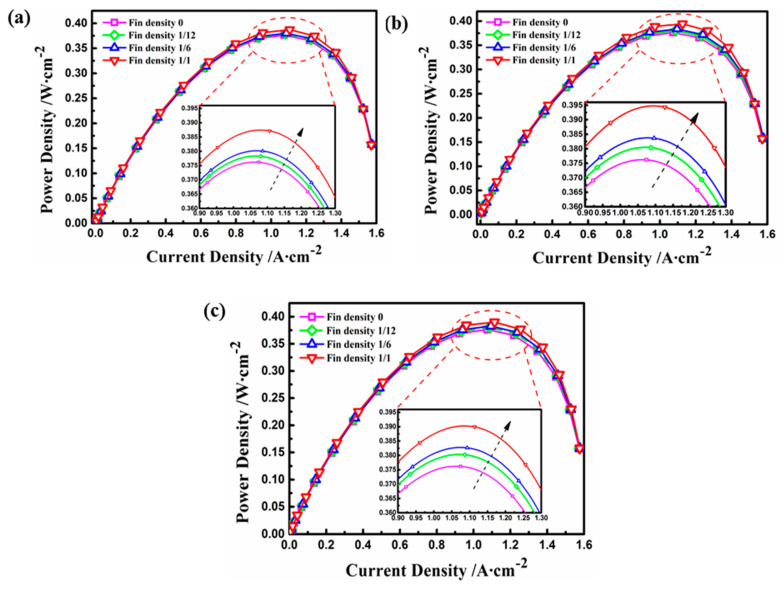
The power densities of different electrode fins: (**a**) Case A with different fin densities; (**b**) Case B with different fin densities; (**c**) Case C with different fin densities.

In order to analyze the quantitative performance of the fuel cell with the electrode fin structure, the current density and output power under different operating conditions were comparatively studied. Figure 8 presents the polarization curves of current density and operating voltage with the various electrode fins. In general, the results indicate that with the decrease in operating voltage, the current density increased gradually. Furthermore, the performance of the fuel cell increased when the density of the electrode fin increased in the same case, and the optimal performance occurred when the density was equal to 1. At an operating voltage of 0.4 V, for instance, the model with a fin density of 1 improved the current density in Cases A to C by 3.3%, 4.2%, and 5.4%, respectively, compared with the basic model. However, under the same fin density, the case with a high aspect ratio had the highest current density when the operating voltage was less than 0.4 V because of the largest contact area under the same electrode surface, and the concentration polarization of Case B was more obvious at the voltage of 0.1 V. Figure 9 shows the influence of different electrode structures on power density. Generally speaking, as the operating voltage increased, the output power for each working condition reached the maximum value under an operating voltage between 0.3 and 0.4 V. The results show that the optimum performance, in terms of power density, was obtained by increasing the electrode fin density and the aspect ratio [27]. Moreover, the maximum values of power density for Cases A, C, and B were 0.387 $Wm^{-2}$, 0.390 $Wm^{-2}$, and 0.395 $Wm^{-2}$, respectively, corresponding to an increase of 4.3%, 5.1%, and 6.5% compared with the maximum power density of 0.371 $Wm^{-2}$ for the basic model.

### 3.4. Overpotential Distribution on the Surface of the Fin

As discussed previously, the fin structure of the porous electrode can improve the performance of the fuel cell by enlarging the surface contact area between the electrode and the GDL. Thus, investigating the voltage loss behavior of the fin structure helps to reveal the mechanism for its electrochemical kinetics performance. With an electrode fin density of 1/12 in the sample cases, the overpotential distribution in the height direction of the fin ($H_{f}$) was compared with the theoretical solution given by Equation (17). The internal resistance of the electrode $R_{s}$ is shown in Table 7, which is composed of mass transfer resistance, ohmic internal resistance, and polarization internal resistance. It can be solved by the slope of the polarization curve.

**Figure 10:**
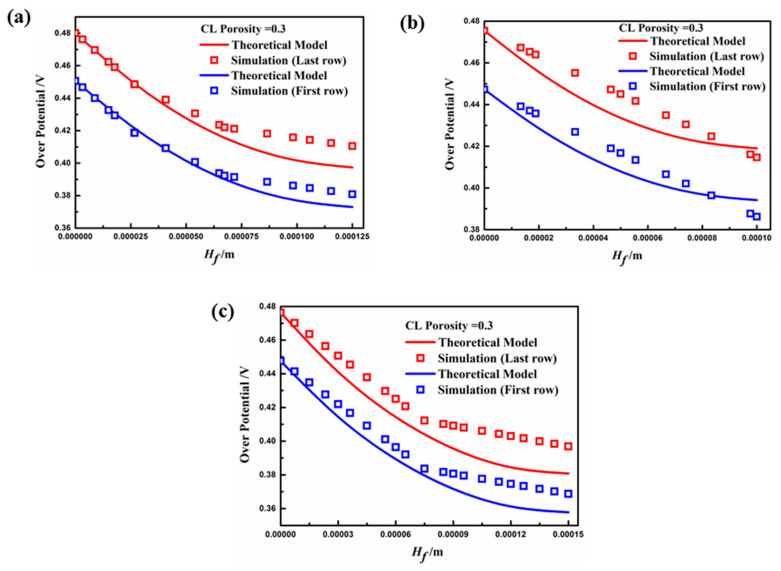
Overpotential distribution in the height direction of the fin ($\varepsilon_{cl}=0.3$, $V_{0}=0.4\;V$): (**a**) Case A; (**b**) Case B; (**c**) Case C.

Figure 10 depicts the distribution of overpotential along the *y*-direction of both the first and last rows of the fin, which coincides well with the mathematical model under the electrode porosity of 0.3. The overpotential gradually decreases along the fin height direction, which is due to the sufficient concentration of the contact reactant at the top of the fin, and the concentration polarization occurred at the base position more obviously. In this figure, the variability between the theoretical model and simulation value is the lowest for Case B, then Case C, and finally Case A. The smaller aspect ratio and larger width of the electrode fin resulted in a non-uniform overpotential distribution along the cross-section of the height direction. Thus, the surface overpotential was smaller than at the central part of the fin molding, leading to a decrease in the current through the electrode surface (the source term). Hence, the overpotential distribution of Case A was almost linear. In addition, the overpotential of the first row of fins was smaller than that of the last row for each case, because the reactants were markedly consumed from the first row to the last row, resulting in insufficient concentrations and polarization.

**Figure 11:**
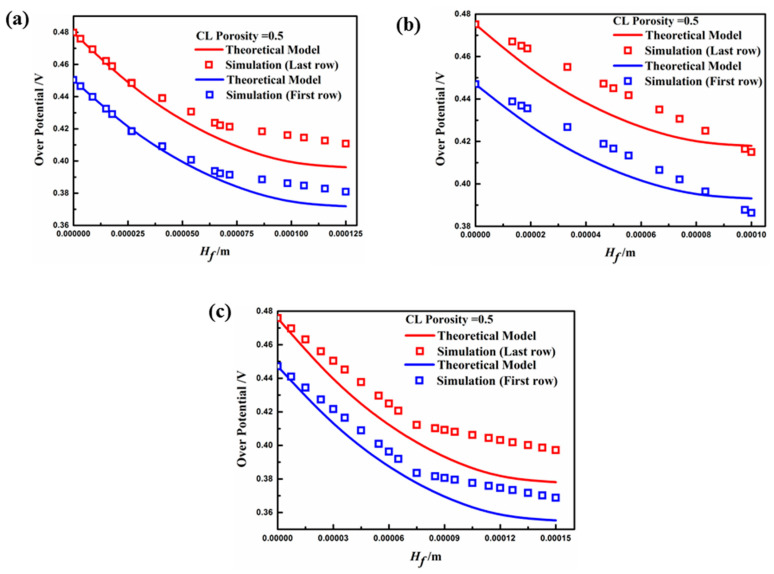
Overpotential distribution in the height direction of the fin ($\varepsilon_{cl}=0.5,\;V_{0}=0.4\;V$): (**a**) Case A; (**b**) Case B; (**c**) Case C.

Figure 11 presents the distribution of overpotential in the *y*-direction of the fin when the porosity of the porous electrode was 0.5. The results show that the overpotential distribution of the simulation results in both the first and last rows of the fin coincides with the theoretical model. However, the electrochemical reaction is slightly enhanced due to the increase in the electrode porosity, which raises the reactant concentration at the contact area on the electrode fin. Thus, the electrode fin in Case C had the best performance when the operating voltage was higher than 0.4 V, due to an especially marked decrease in the average overpotential, which is consistent with previous analyses. It should be noted that, through theoretical model calculations, the effect mechanism of electrode surface overpotential on the performance of fin sizes can effectively explain the kinetic characteristics for different aspect ratios. Furthermore, the fin structure of porous electrodes can be optimized using a suitable model for different PEMFCs in future research.

## 4. Conclusions

In this paper, the design of a novel porous electrode fin-like surface, combined with a theoretical model, significantly impacted the performance of an HT-PEMFC. The effect of fin density and aspect ratio on the performance of the fuel cell was studied, and the conduction mechanism was analyzed using the theoretical model. The results show that the oxygen mole concentration decreased as the number of electrode fins increased, and more oxygen was consumed in Case C than in other cases under the high operating voltage condition. The distribution trend of overpotential in the *y*-direction of the fin coincided well with the theoretical model, particularly in high aspect ratio and narrow fin conditions. The overpotential gradually decreased along the fin height direction and was minimal in the last row. Moreover, the theoretical model concluded that an enlarged contact reaction area for the electrode fin geometry can obtain the optimal performance, consistent with the kinetic characteristics results from the simulations. The research results contribute to the efficient design and preparation of future electrode structures of HT-PEMFCs.

## Figures and Tables

**Table 1:** Geometric parameters of PEM fuel cell.

Parameters	Value
Length of cell, $L_{c}$(m)	0.02
Height of gas channel, $H_{c}$(m)	${1\times 10}^{-3}$ [27]
Gas channel width, $W_{c}$(m)	${7.874\times 10}^{-4}$ [27]
Rib width, $W_{r}$(m)	${9.0932\times 10}^{-4}$ [27]
GDL thickness, $H_{gdl}$(m)	${3.8\times 10}^{-4}$ [27]
Porous electrode thickness, $H_{e}$(m)	${5\times 10}^{-5}$ [28]
Membrane thickness, $H_{m}$(m)	${1\times 10}^{-4}$ [28]

**Table 2:** Source terms.

Source Terms	Expressions
$S_{Am}$	$-\frac{\left|j_{a}\right|}{2F}M_{H_{2}}$
$S_{Cm}$	$-\frac{\left|j_{c}\right|}{4F}M_{O_{2}}+\frac{\left|j_{c}\right|}{2F}M_{H_{2}O}$
$S_{u}$	$-\beta_{F}\left|u\right|u-\left(\frac{\mu }{\kappa }+\frac{S_{m}}{\varepsilon^{2}}\right)u$

**Table 3:** Boundary conditions in the single-channel model.

Parameters	Value
Inlet velocity of anode, $u_{a}\;\;\;\left(m\;s^{-1}\right)$	0.2
Inlet velocity of cathode, $u_{c}\;\;\;\left(m\;s^{-1}\right)$	0.5
Inlet mass fraction of anode $H_{2}$, $w_{H_{2}}$	0.743 [28]
Inlet mass fraction of anode $H_{2}O$, $w_{H_{2}O}$	0.257 [28]
Inlet mass fraction of cathode $O_{2}$, $w_{O_{2}}$	0.228 [28]
Inlet mass fraction of cathode $N_{2}$, $w_{N_{2}}$	0.749 [28]
Inlet mass fraction of cathode $H_{2}O$, $w_{H_{2}O}$	0.023 [28]
Reference pressure, $P\;\;\left(P_{a}\right)$	101,325
Cell temperature, T (°C)	180 [28]

**Table 4:** Physical parameters.

Parameters	Value
GDL porosity, $\varepsilon_{gdl}$	0.4 [36]
Electrode porosity, $\varepsilon_{cl}$	0.3 [37]
Electrolyte porosity, $\varepsilon_{m}$	0.3 [37]
GDL permeability, $\kappa_{gdl}\;\left(m^{2}\right)$	${1.18\times 10}^{-11}$ [28]
Electrode permeability, $\kappa_{cl}\;\left(m^{2}\right)$	${2.36\times 10}^{-12}$ [28]
Electrode conductivity, $\sigma_{cl}$$\left(S\;m^{-1}\right)$	10,000 [38]
GDL electric conductivity, $\sigma_{gdl}$$\left(S\;m^{-1}\right)$	222 [28]
Membrane conductivity, $\sigma_{m}$$\left(S\;m^{-1}\right)$	9.825 [28]
Anode viscosity, $\mu_{a}$$\left(P_{a}s\right)$	${1.19\times 10}^{-5}$ [28]
Cathode viscosity, $\mu_{c}$$\left(P_{a}s\right)$	${2.46\times 10}^{-5}$ [28]
Molar mass of hydrogen, $M_{H_{2}}$$\left(kg\;{mol}^{-1}\right)$	0.002
Molar mass of nitrogen, $M_{N_{2}}$$\left(kg\;{mol}^{-1}\right)$	0.028
Molar mass of water, $M_{H_{2}O}$$\left(kg\;{mol}^{-1}\right)$	0.018
Molar mass of oxygen, $M_{O_{2}}$$\left(kg\;{mol}^{-1}\right)$	0.032
Reference molar concentration of oxygen, $C_{O_{2}\cdot ref}$$\left({mol\;m}^{-3}\right)$	40.88
Reference molar concentration of hydrogen, $C_{H_{2}\cdot ref}$$\left({mol\;m}^{-3}\right)$	40.88

**Table 5:** Simulation cases.

	Case A	Case B	Case C				
$W_{f}\;\;\left(m\right)$	${1\times 10}^{-4}$	${5\times 10}^{-5}$	${1\times 10}^{-4}$	Fin density			
$H_{f}$ $\left(m\right)$	${1\times 10}^{-4}$	${1.25\times 10}^{-4}$	${1.5\times 10}^{-4}$	0	1/12	1/6	1/1
Aspect ratio	1	2.5	1.5				

$\mathrm{Aspect}\;\mathrm{ratio}=H_{f}/W_{f}\;\mathrm{Fin}\;\mathrm{Density}=W_{f}/D_{f}.$

**Table 6:** Grid numbers of different simulation cases.

Fin Density	Case A	Case B	Case C
1/12	1,054,631	1,084,624	1,106,542
1/6	1,145,687	1,174,165	1,194,621
1/1	1,225,984	1,257,431	1,274,165

**Table 7:** $R_{s}$ ($\Omega \cdot {cm}^{2}$) at an operating voltage of 0.4 V.

	Case A	Case B	Case C
$\varepsilon_{cl}=0.3$	0.3327	0.3453	0.3912
$\varepsilon_{cl}=0.5$	0.3282	0.3401	0.3886

## Data Availability

The original contributions presented in this study are included in the article. Further inquiries can be directed to the corresponding authors.

## Nomenclature

L	Length of geometric (m)	α	Electrical transfer coefficient
W	Width of geometric (m)	$\sigma_{A}$	Specific surface area ($m^{-1}$)
H	Height of geometric (m)	θ	Excess potential (V)
u	Fluid velocity ($ms^{-1}$)	η	Overpotential (V)
I	Current density ($Am^{-2}$)	*Subscripts and superscripts*	
F	Faraday constant ($96,487\;C\;{mol}^{-1}$)	gdl	Gas diffusion layer
j	Transition current density ($Am^{-3}$)	r	Rib
M	Molecular weight ($kg\;{mol}^{-1}$)	m	Membrane
$F_{u}$	Force term ($kg/\left(m^{2}s^{2}\right)$)	c	Gas channel
w	Mass fraction (%)	e	Electrode
$x_{k}$	Mole fraction (%)	$H_{2}$	Hydrogen
g	External force ($m\;s^{-2}$)	$O_{2}$	Oxygen
D	Mass diffusivity $\left(m^{2}s^{-1}\right)$	$N_{2}$	Nitrogen
R	Universal gas constant (${8.314\;mol}^{-1}K^{-1}$)	$H_{2}O$	Water
T	Temperature (K)	s	Solid
C	Molar concentration ($mol\;m^{-3}$)	a	Anode
P	Pressure (Pa)	c	Cathode
$E_{eq}$	Equilibrium potential (V)	∞	Bulk condition
$R_{s}$	Internal resistance ($\Omega^{-1}{cm}^{2}$)	o	Standard condition
*Greek symbols*		i	Species index
ρ	Density ($kg\;m^{-3}$)	loc	Location
ε	Porosity	eff	Effective
μ	Dynamic viscosity ($kg\;m^{-1}s^{-1}$)	ref	Reference
κ	Permeability ($m^{2}$)	k	Species index independently of *i*
$\beta_{F}$	Forchheimer drag option (${kg}^{-1}m^{4}$)	cl	Catalytic electrode
σ	Conductivity ($S\;m^{-1}$)	l	Liquid
ϕ	Phase potential (V)	f	Fin
